# Silk fibroin scaffolds seeded with Wharton’s jelly mesenchymal stem cells enhance re-epithelialization and reduce formation of scar tissue after cutaneous wound healing

**DOI:** 10.1186/s13287-019-1229-6

**Published:** 2019-04-27

**Authors:** José E. Millán-Rivero, Carlos M. Martínez, Paola A. Romecín, Salvador D. Aznar-Cervantes, Marina Carpes-Ruiz, José L. Cenis, Jose M. Moraleda, Noemí M. Atucha, David García-Bernal

**Affiliations:** 1 Hematopoietic Transplant and Cellular Therapy Unit, Instituto Murciano de Investigación Biosanitaria-Arrixaca, Virgen de la Arrixaca University Hospital, University of Murcia, Murcia, Spain; 2 Internal Medicine Department, Medicine School, University of Murcia, Avenida Buenavista s/n. El Palmar, Murcia, Spain; 3 Experimental Pathology Unit, Instituto Murciano de Investigación Biosanitaria (IMIB)-Arrixaca, Murcia, Spain; 4 Biotechnology Department, Instituto Murciano de Investigación y Desarrollo Agrario y Alimentario (IMIDA), Murcia, Spain; 5 Physiology Department, Medicine School, University of Murcia, Murcia, Spain

**Keywords:** Mesenchymal stem cells, Wharton’s jelly, Wound healing, Silk fibroin

## Abstract

**Background:**

The treatment of extensive and/or chronic skin wounds is a widespread and costly public health problem. Mesenchymal stem cells (MSCs) have been proposed as a potential cell therapy for inducing wound healing in different clinical settings, alone or in combination with biosynthetic scaffolds. Among them, silk fibroin (SF) seeded with MSCs has been shown to have increased efficacy in skin wound healing experimental models.

**Methods:**

In this report, we investigated the wound healing effects of electrospun SF scaffolds cellularized with human Wharton’s jelly MSCs (Wj-MSCs-SF) using a murine excisional wound splinting model.

**Results:**

Immunohistopathological examination after transplant confirmed the presence of infiltrated human fibroblast-like CD90-positive cells in the dermis of the Wj-MSCs-SF-treated group, yielding neoangiogenesis, decreased inflammatory infiltrate and myofibroblast proliferation, less collagen matrix production, and complete epidermal regeneration.

**Conclusions:**

These findings indicate that Wj-MSCs transplanted in the wound bed on a silk fibroin scaffold contribute to the generation of a well-organized and vascularized granulation tissue, enhance reepithelization of the wound, and reduce the formation of fibrotic scar tissue, highlighting the potential therapeutic effects of Wj-MSC-based tissue engineering approaches to non-healing wound treatment.

**Electronic supplementary material:**

The online version of this article (10.1186/s13287-019-1229-6) contains supplementary material, which is available to authorized users.

## Background

Wound healing is a dynamic and interactive biological response to tissue injury involving a variety of soluble mediators, blood cells, extracellular matrix proteins, and parenchymal cells, in order to repair the skin, to prevent infections, and to restore the tissue integrity and function [1]. This process involves three distinct and overlapping phases: inflammation, cell proliferation (which includes neoangiogenesis, granulation tissue formation, and re-epithelialization), and the remodeling phase (i.e., extracellular matrix remodeling) [2–4]. If the injury compromises the epidermis and dermis, wound healing is characterized by a fibrotic regenerative process, which includes the loss of all skin appendages and formation of a scar. As a result, extensive wounds and chronic wound healing generates life-long disability and a major public health issue, whereas an accurate skin regeneration process can completely restore the original tissue architecture and human health [5].

Standard treatment of chronic wounds is focused on controlling causative factors, but remains unsatisfactory. Mesenchymal stem cells (MSCs) are emerging as a promising candidate for cell-based therapy for the treatment of chronic wounds because of their great potential to enhance tissue repair and regeneration after injury [6–9].

From the different available sources to obtain MSCs, extraembryonic tissues such as the umbilical cord Wharton’s jelly (Wj) represents a cost-effective, feasible, and non-invasive method to isolate MSCs and is being considered advantageous compared to MSCs obtained from adult tissues (i.e., bone marrow or adipose tissue). Among other properties, Wj-MSCs have demonstrated higher proliferative capacity with no signs of senescence over serial passages compared to bone marrow MSCs [10–12]. Wj-MSCs have shown to be less immunogenic than their adult tissues counterparts and also possess potent immunomodulatory properties due to the release of large amounts of anti-inflammatory molecules such as TGFβ, IL-10, IDO, TSG-6, and PGE_2_ compared to bone marrow MSCs [13, 14].

The systemic and local delivery of MSCs has shown to be effective for wound healing. Although systemic delivery of MSCs allows cells to reach the injury site, cell engraftment and survival after administration are limited [15, 16]. Thus, novel cell delivery systems to enhance wound healing are needed [17].

Several biosynthetic scaffolds have been used alone or in combination with cells to treat wounds. Among them, silk fibroin (SF) cellularized with MSCs from different sources has been shown to be effective in repairing experimental skin wounds in previous models [18–20].

Electrospun SF scaffolds have two distinctive characteristics that make them very appropriate for tissue engineering: the morphology and architecture of the electrospun structure are similar to those of natural extracellular matrix (ECM), and the scaffold structure changes dynamically over time as the polymer nanofibers degrade, allowing the seeded cells to proliferate and produce their own ECM [21].

Here, we have used an excisional wound splinting model to create non-healing wounds in hairless SKH1 mice in order to investigate if administration of human Wj-MSCs on SF scaffolds into the wound bed could benefit the skin regenerative process. After characterizing the Wj-MSC-cellularized SF scaffolds, as well as the in vitro mesenchymal properties of the cells, we asked the following questions: (i) do SF scaffolds possess any biologic effect on the course of the wound healing process? (ii) does the Wj-MSC local administration (w/o the SF scaffold) induce a similar effect on the wound healing compared to the Wj-MSC-based SF construct? and (iii) is there any synergistic effect on the wound healing process when the Wj-MSCs are injected locally on the wound edge together with the application of the Wj-MSCs-SF construct?

## Methods

### Elaboration of electrospun silk fibroin scaffolds

Cocoons were obtained from *Bombyx mori* silkworms, chopped into four or five pieces, and boiled in 0.02 M Na_2_CO_3_ solution for 30 min to remove the glue-like sericin proteins, following previously described protocols [22]. Briefly, the extracted silk fibroin (SF) was dissolved in 9.3 M LiBr (Acros Organics) for 3 h at 60 °C, to generate a 20% solution that was dialyzed against distilled water for 3 days (Snakeskin Dialysis Tubing 3.5 KDa MWCO, Thermo Scientific) and concentrated by dialysis against 30% polyethylene glycol for 24 h at 10 °C to obtain 19–20% regenerated SF solutions that were used for subsequent electrospinning experiments. For electrospinning, a voltage of 19–21 kV was applied to the capillary tube, the distance between the tip of the tube and the collector was adjusted to 44 cm, and the selected injection rate of the polymer solution was 1 ml/h. After fabrication, the electrospun meshes were annealed by immersion in a bath of absolute methanol for 45 min to induce a structural transition from an amorphous (random coil) to a β-sheet conformation. After, the SF meshes were cut into 10-mm-diameter disks, disinfected with 70% aqueous ethanol solution and left under the UV-C germicidal lamp to ensure sterilization of the patches.

### Isolation and characterization of Wj-MSCs

Umbilical cord donors provided written and informed consent according to the guidelines of the Ethics Committee of our institution (Hospital Clinico Universitario Virgen de la Arrixaca, Murcia, Spain). Human Wj-MSCs were isolated by the explant method as previously described [23, 24]. Briefly, each cord was sectioned into 3–5-cm-long pieces, amnion was cut along the horizontal axis, and blood vessels with blood clots inside were removed. Then, cord pieces were placed with the inside faced to the bottom of a sterile 10-cm^2^ petri dish. Explants were left to attach to the plate, and complete culture medium (DMEM supplemented with 15% fetal bovine serum, 1% l-glutamine, and 1% penicillin/streptomycin (all from Life Technologies)) was added. Finally, the MSCs adhering to the plate were grown up to 80–90% confluence and submitted to serial diluted passages.

Wj-MSCs were analyzed by flow cytometry to confirm their mesenchymal phenotype. Briefly, cells were incubated with fluorescence-conjugated specific monoclonal antibodies for CD73, CD90, CD105, CD14, CD20, CD34, CD45, CD80, CD86, and HLA-DR (Miltenyi Biotec) for 30 min at 4 °C in the dark. Specific isotype monoclonal antibodies were used to exclude non-specific staining. After labeling and washing, cells were acquired using a BD FACSCanto flow cytometer (BD Biosciences) and analyzed with Kaluza analysis software (Beckman Coulter).

Regarding the immunological properties, we analyzed the effect of Wj-MSCs on the proliferation of human peripheral blood mononuclear cells (MNCs). In brief, 1 × 10^5^ responder MNCs were cultured for 5 days in 24-well plates with αCD3αCD28 beads (Dynabeads® Human T-Activator CD3/CD28) (Thermo Fisher Scientific) alone or in combination with different ratios of human bone marrow (BM) or Wharton’s jelly (Wj)-derived MSCs. On day 5, cultures were pulsed with [^3^H]thymidine ([^3^H]TdR, Amersham) for 18 h. After, cells were harvested onto glass fiber filters, and radionuclide uptake was measured using a micro β-liquid scintillation counter. All experiments were performed in triplicate.

For multipotent differentiation assays, Wj-MSCs were differentiated toward the adipogenic, osteogenic, and chondrogenic lineages using StemMACS™ AdipoDiff, OsteoDiff, and ChondroDiff differentiation media (Miltenyi Biotec), following the manufacturer’s instructions. After, adipogenic differentiation was evaluated using Oil Red O staining (Sigma-Aldrich). For osteogenic differentiation, cells were stained with Alizarin Red and SigmaFast™ BCIP-NBT (both from Sigma-Aldrich). Finally, chondrogenic differentiation was assessed by staining with Alcian blue (Sigma-Aldrich). All experiments were performed in triplicate.

To analyze the ability of Wj-MSCs to produce extracellular matrix (ECM) proteins (i.e., collagen), a Masson’s trichrome was performed in vitro on a silk fibroin scaffold cultured with Wj-MSCs for 4 days by using a commercial staining kit (Masson’s trichrome with Aniline blue, Bio-Optica) and following the manufacturer’s recommendations. After the staining, the Wj-MSC-cellularized scaffold was mounted on a slide and examined with a standard microscope (Carl Zeiss Axio Scope A10). Using this histochemical procedure, collagen deposition can be identified as a light-blue staining.

### Electrospun SF scaffolds and Wj-MSCs

Pieces of electrospun SF (1-cm-diameter disks) were placed into 24-well cell culture plates, used as scaffolds for culturing 4 × 10^4^ cells per well, and kept in an incubator during 4 days until surgery or in vitro experiments.

To analyze possible phenotypic changes in the expression of mesenchymal markers, cells were analyzed by flow cytometry as described above.

Apoptosis analysis by using Annexin-V Apoptosis Detection Kit (BD Bioscience) was carried out to discard possible cytotoxic effects of SF scaffolds on Wj-MSCs. At first, MSCs were seeded at a density of 1.5 × 10^4^ cells/cm^2^ on the SF scaffolds or the plastic bottom (as a positive control) in 48-well plates, maintained in complete culture medium up to 10 days, stained with PE-conjugated Annexin-V and 7-AAD, and analyzed by flow cytometry. All determinations were performed in triplicate.

### Mouse excisional wound splinting model

Eight- to 12-week-old hairless SKH1 mice (Charles River) were used. All techniques, protocols, and animal proceedings employed were performed according to the Animal Ethics Committee of University of Murcia following the Spanish and European governmental regulations on the use of animals for scientific research (Directive 86/609/EC (RD223/1998, RD1201/2005, LAW32/2007) and Directive 2010/63/EU (RD53/2013, LAW 6/2013, Commission Recommendation 2007/526/EC)).

The mouse excisional wound splinting model was carried out as previously described with some modifications [25, 26]. Firstly, mice were individually anesthetized, and then, two donut-shaped silicone wound splints (Grace Bio-Labs) were fixed to the skin on each side of the dorsal surface midline. Next, a 10-mm-diameter full-thickness wound was made aseptically within the splint, and the stem cells were used according to the experimental protocol, i.e., injected into the edge of the wound or applied through cellularized SF patches. In all cases, an occlusive sterile adhesive membrane (Oper film, IHT) was used to completely cover the wounds and splints. In the untreated group of animals, and to avoid possible secondary infections, wounds were covered with Linitul® (Bama-Geve SLU), a dressing frequently employed in the clinical practice which is indicated to treat wounds, bedsores, ulcers, and varicose. It is composed of 18.5 mg of balsam of Peru and 167.8 mg of castor oil per gram.

Two groups of 75 mice were experimentally related to two different umbilical cord donors. Both groups were divided into five different subgroups (*N* = 15): (i) wounds covered by Linitul, (ii) wounds covered by SF patches, (iii) Wj-MSCs injected at the edge of the wound (1 × 10^6^ cells in 100 μl PBS), (iv) wounds covered by cellularized SF scaffold (5 × 10^4^ cells seeded onto the scaffold for 4 days before surgery), and (v) wounds treated with Wj-MSCs injected at the edge (1 × 10^6^ cells in 100 μl PBS) and also cellularized SF patches (5 × 10^4^ cells seeded onto the scaffold for 4 days before surgery). Every subgroup underwent the sacrifice of three mice at different times, i.e., 48 h and 7, 14, 21, and 28 days.

### Histological and immunohistochemical study of wound healing

Skin samples from all groups were fixed in 4% neutral buffered formalin (Panreac Quimica S.A.) for 24 h. Samples were then processed, paraffin embedded, and sectioned (3 μm thick). Sections were stained with a standard hematoxylin and eosin (H&E) staining procedure for histopathological evaluation. Additionally, an indirect ABC immunohistochemical procedure was performed for immunohistopathological analysis, by using a commercial kit (EnVision Flex, Dako) according to the manufacturer’s recommendations and using different antibodies according to the study.

Polymorphonuclear neutrophils (PMNs), macrophages, and T lymphocytes were identified by standard histopathological analysis by their morphological features (segmented nuclei) (i.e., PMNs), or after immunostaining using an anti-F4/80 (AbD Serotec) for macrophages, and an anti-CD3 antibody (Dako) for T lymphocytes, respectively. The average numbers of positive cells were determined by counting such cells in ten high-power fields (HPF).

Histological evaluation of the wound healing included measuring of angiogenesis and fibrogenesis by CD31 (Dako) or alpha-smooth muscle antigen (αSMA, Dako) and desmin (Abcam) immunostaining, respectively. In order to establish the degree of angiogenesis, three samples of each experimental group were chosen to make ten HPF of the most vascularized zones. The total area of vascularization was then measured in each image. The final result was expressed as the median vascularized surface expressed in square micrometers per field. For quantitative assessment of myofibroblast proliferation, the number of positive cells for α-SMA and desmin expression was determined at ten random HPF from three different samples of each group. Result was established as the median number of positive cells per field. Finally, to establish the capacity of Wj-MSCs to transdifferentiate into keratinocyte-like cells, an immunostaining using a specific anti-human pan cytokeratin antibody (Abcam) was also performed. All immunohistopathological analyses were performed by using a standard light microscope (Zeiss Axio Scope A10, Carl Zeiss) with a digital camera (Axio Cam IcC3, Zeiss) and a specific digital analysis software (Axio Vision ver. 4.8, Zeiss).

The presence of hWJ-MSCs in the wounds was assessed by immunohistochemistry using a specific anti-human CD90 antibody (Abcam). According to the manufacturer’s specifications, this antibody does not react with mouse cells. The positive immunoreaction of this antibody against hWJ-MSCs was previously tested on formalin-fixed and paraffin-embedded pellets of such cells.

### Statistical analysis

All statistics were performed using the GraphPad Prism software package v6. Results are presented as the mean ± standard deviation. To test for statistical significance, the Mann-Whitney test or one-way ANOVA followed by Bonferroni’s post hoc comparison tests was used. Results were considered to be significant if the *p* value was equal to or less than 0.05.

## Results

### In vitro characterization of Wj-MSCs

As reported previously, mesenchymal stem cells isolated from the umbilical cord (i.e., Wj-MSCs) by the explant method displayed a typical fibroblastic, spindle-shaped morphology in culture comparable to that of other sources of mesenchymal stem cells (not shown) [27–29].

**Fig. 1:**
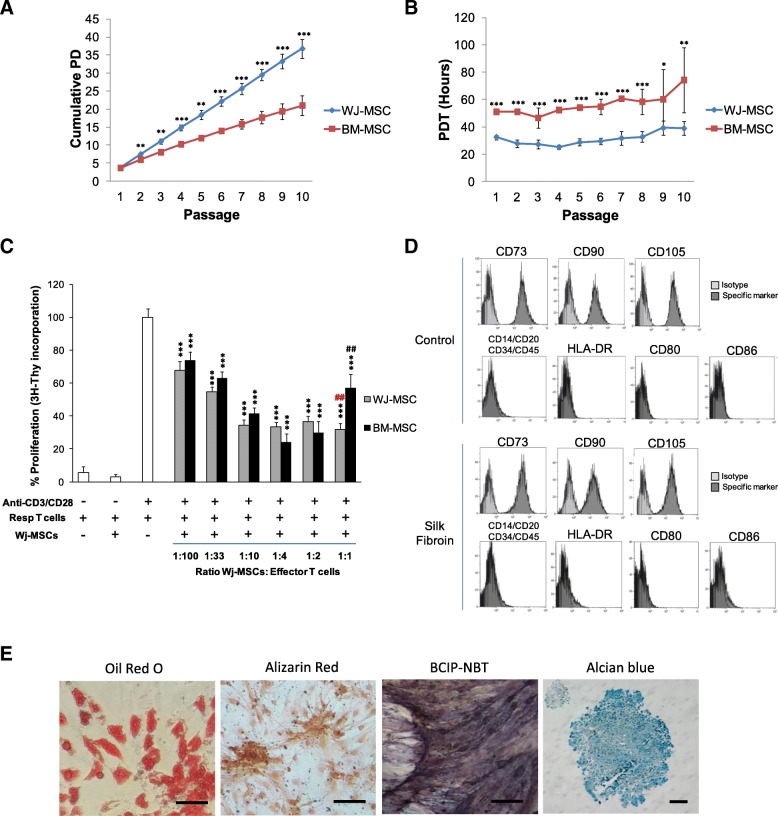
Proliferation kinetics of Wj-MSCs and BM-MSCs overextended in vitro propagation. **a** Number of cumulative population doubling level (PD) as a function of time in culture. **b** Cell population doubling time (PDT) (hours) after sequential passages. Statistically significant differences using one-way ANOVA, *N* = 3; *p < 0.05, **p < 0.01, ***p < 0.001. **c** The capacity of BM- or Wj-MSCs to inhibit the proliferation of stimulated peripheral blood T cells was analyzed. BM- or Wj-MSCs were cultured with 1 × 10^5^ MNCs in different ratios and stimulated with CD3/CD28 beads for 5 days. After, the proliferation of the T cells was measured by thymidine (^3^H-Thy) incorporation. MSCs from both sources significantly inhibited the proliferation of T cells in a dose-dependent manner (***p < 0.001). The proliferation of T cells in the presence of Wj-MSCs was significantly lower than that obtained using BM-MSCs at the same ratio (^##^*p* < 0.01). All data are presented as mean ± SD. *N* = 3. **d** Immunophenotypical analysis of Wj-MSCs by flow cytometry. Wj-MSCs were seeded at a density of 5 × 10^4^ cells/cm^2^ on plastic culture plates (control) (upper panels) or SF patches (bottom panels) for 4 days. After, cells were detached and labeled with specific antibodies for the indicated markers or their control isotypes. Histograms show representative flow cytometry results obtained from *N* = 3 independent experiments. **e** To evaluate Wj-MSCs multipotent differentiation properties, cells were cultured in adipogenic, osteogenic, and chondrogenic differentiation media for 14–21 days. After, differentiation was evaluated by staining of lipid droplets with Oil Red O (adipogenic, right), by detection of calcium depositions and alkaline phosphatase activity by Alizarin Red and BCIP-NBT staining (osteogenic, middle left and middle right, respectively), or by detection of glycosaminoglycans by Alcian blue staining (chondrogenic, right). Images shown are representative of *N* = 3 independent experiments. Scale bar 200 μm. Abbreviations: PD population doubling level, PDT population doubling time, Wj-MSCs Wharton’s jelly MSCs, BM-MSCs bone marrow MSCs

The proliferation and growth efficiency of Wj-MSCs were determined by analysis of the cumulative population doubling level (PD) and population doubling time (PDT) and compared to that displayed by bone marrow counterparts (i.e., BM-MSCs). As shown in Fig. 1a, the mean cell yield was significantly higher for Wj-MSCs compared to BM-MSCs (1.9-fold, **p < 0.01) and related to a relatively constant number of about 3.7 PD per passage accumulating to a total of 37 PD (vs. 21 PD for BM-MSCs) in the 2-month culture period. Also, Wj-MSCs had a significant shorter PDT compared to BM-MSCs in every single passage (from P1 up to P8, ***p < 0.001; P9, *p < 0.05; P10, **p < 0.01) (Fig. 1b). Therefore, Wj-MSCs displayed an increased proliferative capacity compared to BM-MSCs.

To assess the immunomodulatory properties of Wj-MSCs, we performed in vitro co-cultures with CD3/CD28-stimulated human peripheral blood T cells and Wj-MSCs or BM-MSCs at different ratios. As shown in Fig. 1c, both Wj-MSCs and BM-MSCs inhibited the proliferative response of CD3/CD28-stimulated T cells in a dose-dependent manner (***p < 0.001 for all ratios of MSC to T cells compared to anti-CD3/CD28-stimulated T cells alone). Interestingly, the inhibition of proliferation mediated by Wj-MSCs was slightly higher than that observed for BM-MSCs at ratios 1:100, 1:33, or 1:10, although not statistically significant. However, Wj-MSC-mediated inhibition of proliferation was significantly higher than that achieved for BM-MSCs at ratio 1:1 (Wj-MSC 32 ± 3% proliferation vs. BM-MSC 57 ± 5% proliferation, ^##^*p* < 0.01).

Flow cytometry immunophenotyping studies showed that the MSC surface markers CD73, CD90, and CD105 were expressed to levels greater than 99.5%, whereas expression of the hematopoietic markers CD14, CD20, CD34, and CD45; the MHC-class II HLA-DR; or the co-stimulatory molecules CD80 and CD86 were lower than 5% (Fig. 1d, upper panels).

Also, we evaluated the multilineage differentiation potential of Wj-MSCs into mesodermal lineages after culturing cells in specific adipogenic, osteogenic, and chondrogenic differentiation media (Fig. 1e). Wj-MSCs displayed accumulation of lipid vacuoles (adipocytes, Oil Red O staining, Fig. 1e), formation of mineral depositions and alkaline phosphatase activity (osteoblasts, Alizarin Red and BCIP-NBT staining, Fig. 1e), and expression of glycosaminoglycans (chondroblasts, Alcian blue staining, Fig. 1e).

### Characterization of physical and mechanical properties of electrospun SF scaffolds

The SEM pictures of the SF meshes show the fibers produced from regenerated SF solutions to be cylindrical and uniform throughout their lengths (Additional file 2: Figure S1A). The average value of the diameter of the electrospun fibers was 2417 ± 547 nm (Additional file 2: Figure S1B). No significant differences were found between replicates of the study, which shows a consistent production method. Average values of Young’s modulus (MPa) (51.8 ± 8.5), the elongation at break (%) (4.4 ± 1.1), and the ultimate strength (MPa) (1.4 ± 0.5) were calculated using stress-elongation curves generated from tensile tests. There were no significant differences in the mechanical properties between the three replicates tested for each experimental condition (Additional file 2: Figure S1C). Based on the morphology and mechanics of the scaffold, it was suggested that the electrospun nanofibrous structure could represent a suitable skin substitute (Additional file 1).

### Electrospun SF scaffolds cellularized with Wj-MSCs

**Fig. 2:**
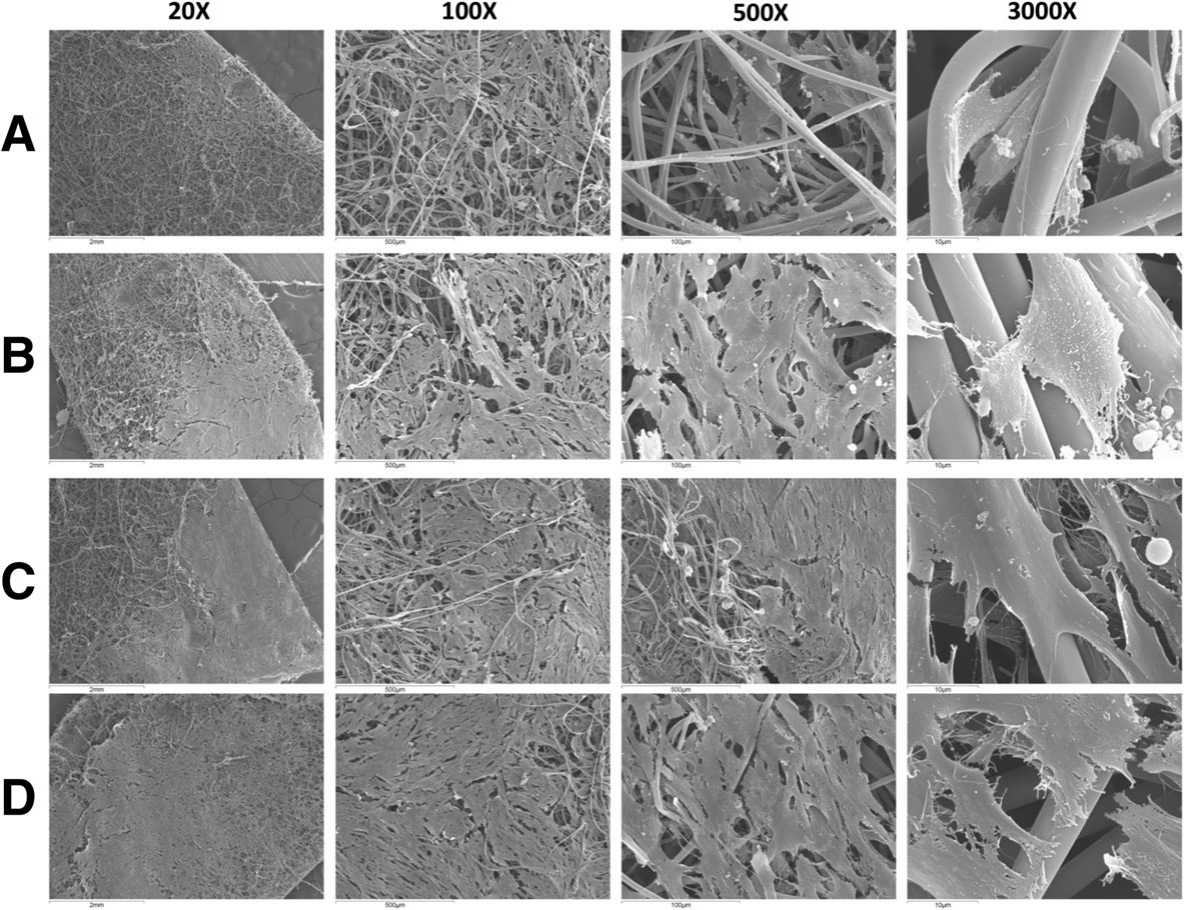
Scanning electron microscopy micrographs of Wj-MSCs growing on electrospun SF scaffold at different cell densities. SF patches were seeded at a density of 3 × 10^4^cells/cm^2^ (**a**), 4 × 10^4^cells/cm^2^ (**b**), 5 × 10^4^cells/cm^2^ (**c**), and 6 × 10^4^cells/cm^2^ (**d**) and cultured for 4 days. Images at a magnification of × 20, × 100, × 500, and × 3000 are shown. *N* = 3

SF scaffolds kept their integrity and three-dimensional structure after seeding with Wj-MSCs (Fig. 2a–d). The Wj-MSCs adhered and spread on the surface of the SF fiber network, migrated through the pores and grew under layers of the fiber network. Also, cells interacted and integrated well with the surrounding fibers. Cells grew in the direction of fiber orientation, forming a three-dimensional and multicellular network according to the architecture of the scaffold. Analysis of the images revealed that Wj-MSCs seeded at intermediate densities (i.e.*,* 5 × 10^4^ cells/cm^2^) (Fig. 2c) formed a continuous monolayer on the SF scaffold but leaving some spaces available for further growth and avoiding an early confluence.

Apoptosis analysis revealed that Wj-MSCs cultured on SF patches (i.e., Wj-MSCs-SF) displayed a significant decrease in viability over the first 3 days after seeding, but increased progressively to values > 80% from day 5 onward (Additional file 3: Figure S2A-B). Although the percentage of viable cells in the Wj-MSCs-SF group was significantly lower than that in the control group during the first 5 days of culture, no differences between them were detected in later days. Also, Wj-MSCs cultured on SF patches for 4 days maintained their mesenchymal immunophenotypical profile (Fig. 1d, bottom panels).

Since collagen is one of the main structural components of the dermis, we evaluated if Wj-MSCs seeded into SF scaffolds were able to express and/or secrete this ECM component. Masson’s trichrome staining of SF scaffolds cellularized with Wj-MSCs displayed a slight collagen deposition (light-blue staining) into the SF-crosslinked nanofibers (Additional file 4: Figure S3A-B).

In summary, SF patches could be considered as a biocompatible scaffold for Wj-MSCs since it may preserve its proliferation, mesenchymal immunophenotype, ECM protein expression, and long-term cell viability.

### Histological and immunohistochemical study of wound healing

**Fig. 3:**
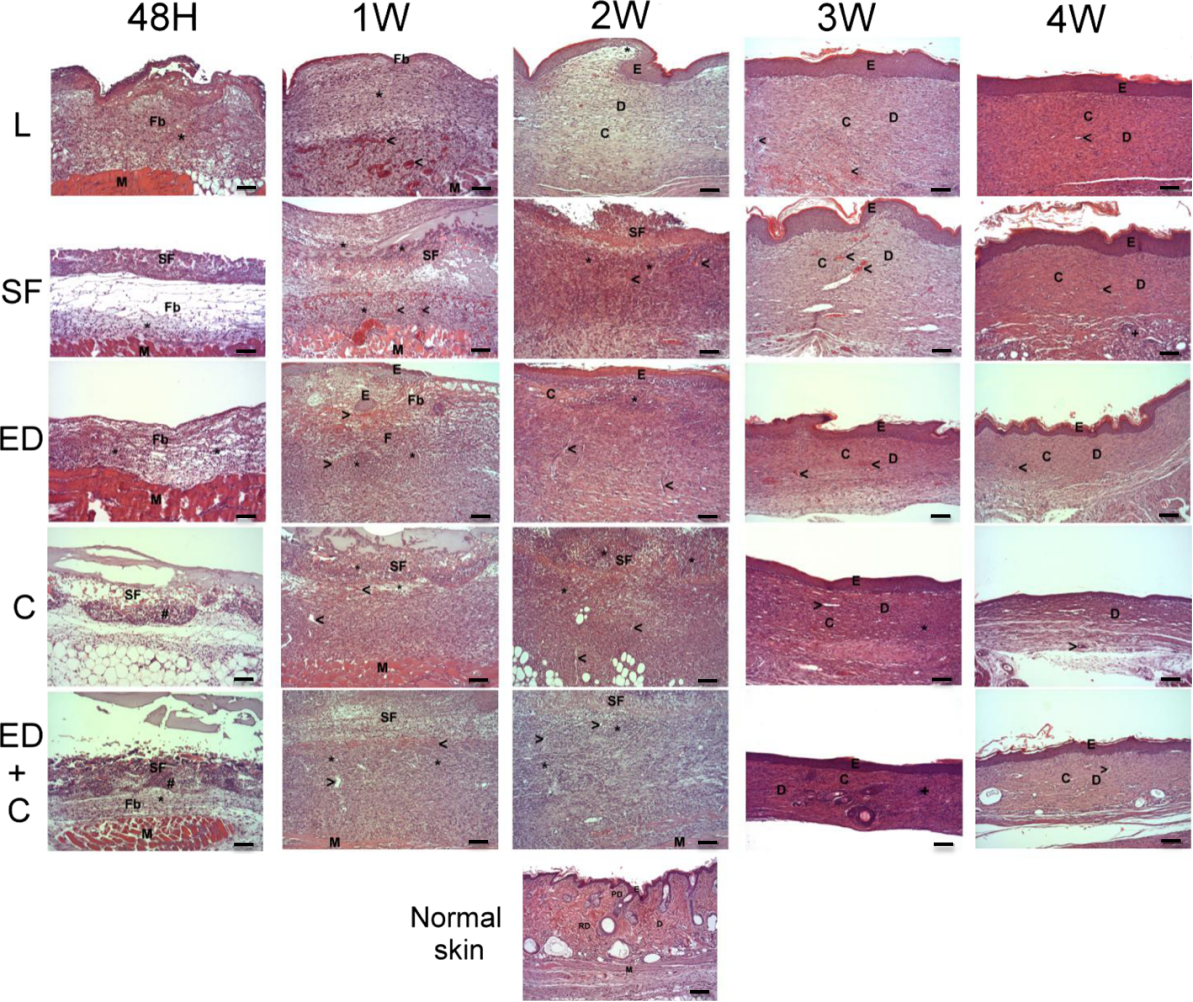
Histopathological assessment of wound healing by hematoxylin and eosin (H&E) staining. Control groups: *L* (wounds covered with Linitul) and *SF* (wounds covered with SF scaffolds). Treated groups: *ED* (wounds treated with Wj-MSCs injected at the edge), *C* (wounds covered with SF patches cellularized with Wj-MSCs), and *ED+C* (wounds with SF patches cellularized with Wj-MSCs over the wound bed combined with Wj-MSCs injected at the edge). Abbreviations: *Fb* fibrin, “*” inflammatory infiltrate and edema, “>” blood vessels, *E* epidermis, *M* muscular layer, *D* dermis, *C* collagen, *SF* silk fibroin scaffold, *PD* papillary dermis, *RD* reticular dermis. Scale bar 100 μm

A chronologic histological and immunohistochemical analysis of wound samples, regarding inflammation, re-epithelialization, granulation tissue formation, angiogenesis, and remodeling of the wound, was carried out to determine the changes produced in situ after the inclusion of the SF scaffolds and/or Wj-MSCs during the skin regeneration process (Fig. 3).

The histopathologic findings of skin wound sections, regarding re-epithelialization and final configuration of the dermal layer, were comparable in Linitul (i.e., the untreated group with wounds only covered by Linitul) and SF groups (i.e., wounds covered by SF patches, w/o Wj-MSCs), although with some temporary differences. In the Linitul group (L), the re-epithelialization was completed after 2 weeks, and the wound healing process finished with the formation of a scar, characterized by a hyperplastic epidermis and a dermis composed by a dense and regular connective tissue. In the SF scaffold-treated group (SF), the re-epithelialization was completed with a hyperplastic epithelial layer around the third week of healing, and at the end of the wounding process, the dermis was mainly composed of well-formed dense connective tissue with a regular configuration. Besides, in Wj-MSCs-Edge group (ED) (i.e., Wj-MSCs injected at the edge of the wound, w/o SF scaffold), the granulation tissue observed at week 2 was quite similar to that observed at week 1 in the Linitul group. In any case, the combination of SF scaffold and Wj-MSCs (C) (i.e., wounds covered by cellularized SF scaffold) induced a delay in the wound healing process, which ends with a scar similar to that observed with Linitul. Finally, the combined treatment of cellularized SF scaffold and Wj-MSCs injected into the wound edge (ED+C) displayed a collagen dermis organization that was more similar to that typically observed in the normal skin of hairless SKH1 mice.

### Analysis of leukocyte infiltrates

**Fig. 4:**
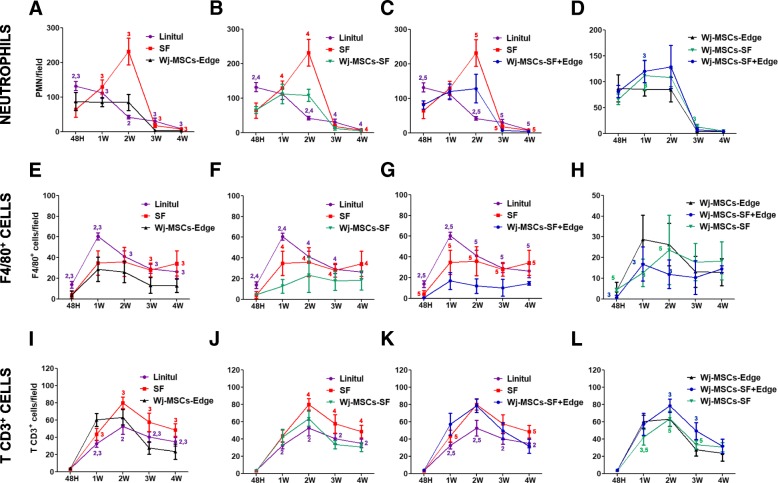
Influx of inflammatory cells into wounded tissues: polymorphonuclear neutrophils (**a**–**d**), macrophages (**e**–**h**), and T cells (**i**–**l**). Experimental groups: wounds covered with Linitul (purple line), wounds covered with SF scaffolds (red line), Wharton’s jelly MSCs injected at the wound edge (Wj-MSCs-Edge) (black line), wounds covered with SF scaffolds cellularized with Wharton’s jelly MSCs (Wj-MSCs-SF) (green line), and wounds treated with Wharton’s jelly MSCs injected at the wound edge and cellularized SF scaffolds onto the wound bed (Wj-MSCs-SF+Edge) (blue line). Statistically significant differences (*p* < 0.05) compared to Linitul (value 1), scaffold (value 2), Wj-MSCs-Edge (value 3), Wj-MSCs-SF (value 4), and Wj-MSCs-SF+Edge (value 5), according to one-way ANOVA

In order to establish differences in the inflammatory infiltration between the different groups of animals, an immunophenotypical characterization of such infiltrate was performed. Regarding polymorphonuclear neutrophil (PMN) infiltrate, high numbers of this subpopulation were observed in the SF scaffold group within the first 2 weeks, but when the Wj-MSCs were associated to the scaffold or injected in the wound edges (i.e., Wj-MSCs-SF, Wj-MSCs-Edge, and Wj-MSCs-SF+Edge), their number was significantly reduced (Fig. 4a–d). Similar behavior was also observed with the macrophage (i.e., F4/80^+^ cells) infiltrate. Again, a significant lower count of this leukocyte subpopulation was observed in the Wj-MSC-treated groups (Fig. 4e–h). Regarding T CD3^+^ cell infiltrate, a peak of this subpopulation was reached at week 2 in all groups (Fig. 4i–l). Higher cell numbers were observed in the SF scaffold group, but newly, there was a decrease in T cell counts in the Wj-MSC-treated groups. Therefore, the Wj-MSCs diminished both innate and adaptative immune infiltrates compared to the groups treated without cells.

### Assessment of angiogenesis by expression of the endothelial marker CD31 in the wound sites

The degree of vascular infiltration was measured by analysis of CD31 expression in the wounds.

**Fig. 5:**
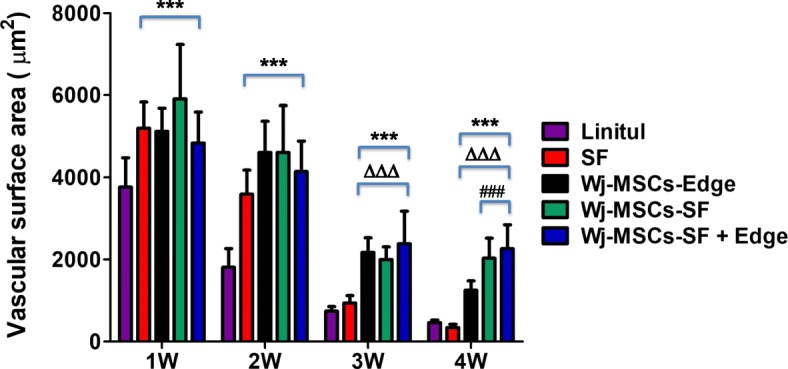
Vascular surface area (expressed in square micrometers per field) determined by immunohistochemical analysis of CD31 expression in wound sections. Vascularized area was significantly increased compared to the untreated group (Linitul) (***p < 0.001), SF group (^∆∆∆^*p* < 0.001), or Wj-MSCs-Edge-treated group (^###^*p* < 0.001), respectively, according to one-way ANOVA. Results are shown as mean ± SD of the most vascularized areas measured in three different mice of each group, corresponding to images made at × 400 magnification. Experimental groups: SF (wounds covered with silk fibroin scaffold), Wj-MSCs-Edge (Wj-MSCs injected at the wound edge), Wj-MSCs-SF (wounds covered with silk fibroin scaffold cellularized with Wj-MSCs), and Wj-MSCs-SF+Edge (wounds treated with Wj-MSCs injected at the wound edge and cellularized silk fibroin scaffold onto the wound bed)

During the first 2 weeks after wound, all experimental treated groups (i.e., SF, Wj-MSCs-Edge, Wj-MSCs-SF, and Wj-MSCs-SF+Edge) displayed a significant increased vascular surface area compared to untreated (i.e., Linitul) animals (***p < 0.001) (Fig. 5). However, at the third week and onwards, the wound vascularized area was decreased in the Linitul and SF scaffold groups (i.e., w/o Wj-MSCs), whereas in all the Wj-MSC-treated groups, the vascularized area was significantly higher than that observed in the absence of Wj-MSCs (^∆∆∆^*p* < 0.001). Remarkably, at the later time point studied (i.e., 4 weeks after wounding) and compared to the group of animals receiving Wj-MSCs only injected into the wound edge (Wj-MSCs-Edge), it was observed that there was a significant increased vascular surface area in the Wj-MSCs-SF and Wj-MSCs-SF+Edge groups (^###^*p* < 0.001). These results suggest that Wj-MSC-based cell therapy in combination with the SF scaffold may contribute to the stimulation of neoangiogenesis in the wounded tissue.

### Expression of α-smooth muscle actin and desmin during wound healing

**Fig. 6:**
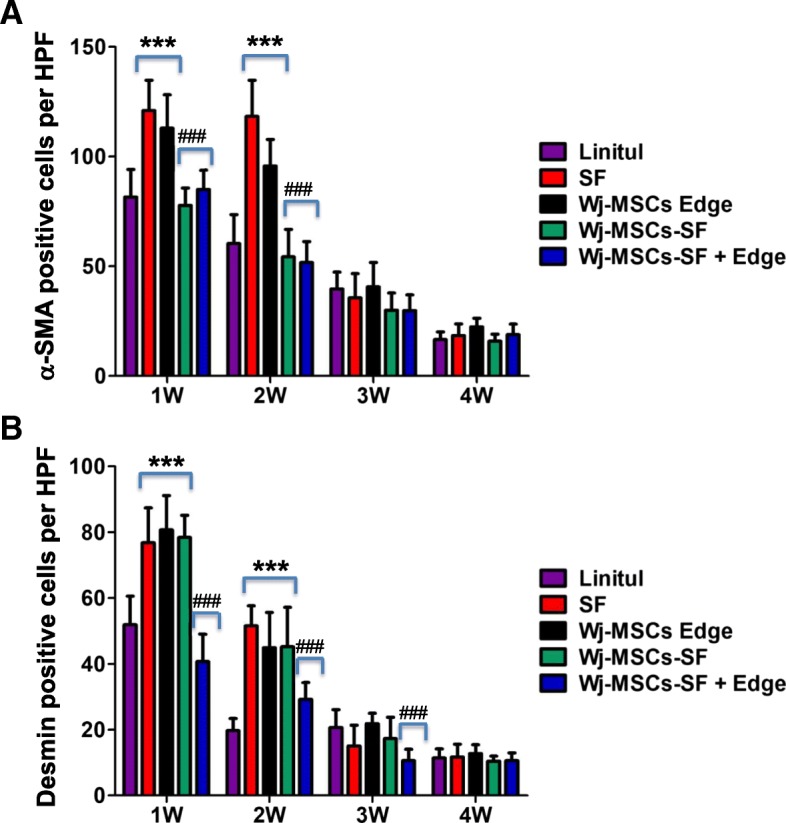
Immunohistochemical analysis of α-SMA expression and desmin in wound sections. For quantitative assessment of myofibroblast proliferation, the number of positive cells for α-SMA (**a**) or desmin (**b**) was determined in ten random sections at × 400 magnification from three different mice of each group. Results are shown as mean ± SD. Myofibroblast proliferation was significantly increased compared to the untreated group (Linitul) (***p < 0.001) or significantly decreased compared to the Wj-MSCs-Edge-treated group (^###^*p* < 0.001), respectively, according to one-way ANOVA. Experimental groups: SF (wounds covered with silk fibroin scaffold), Wj-MSCs-Edge (Wj-MSCs injected at the wound edge), Wj-MSCs-SF (wounds covered with silk fibroin scaffold cellularized with Wj-MSCs), and Wj-MSCs-SF+Edge (wounds treated with Wj-MSCs injected at the wound edge and cellularized silk fibroin scaffold onto the wound bed)

The immunohistochemical analysis of α-smooth muscle actin (α-SMA) and desmin expression, both markers predominantly expressed in myofibroblasts, revealed that the cell therapy using Wj-MSCs-SF and Wj-MSCs-SF+Edge gave rise to a lower myofibroblast proliferation into the wound within the first 2 weeks of treatment compared to that observed in the SF scaffold or Wj-MSCs-Edge groups (Fig. 6). At the third and fourth weeks, a very low α-SMA and desmin expression was observed in the combined therapy groups (i.e., Wj-MSCs-SF and mainly Wj-MSCs-SF+Edge) than all other groups. These results point that the combined therapy using Wj-MSCs and the SF scaffold reduces the myofibroblast proliferation.

### Expression of the mesenchymal stem cell marker CD90 on the wound sites

**Fig. 7:**
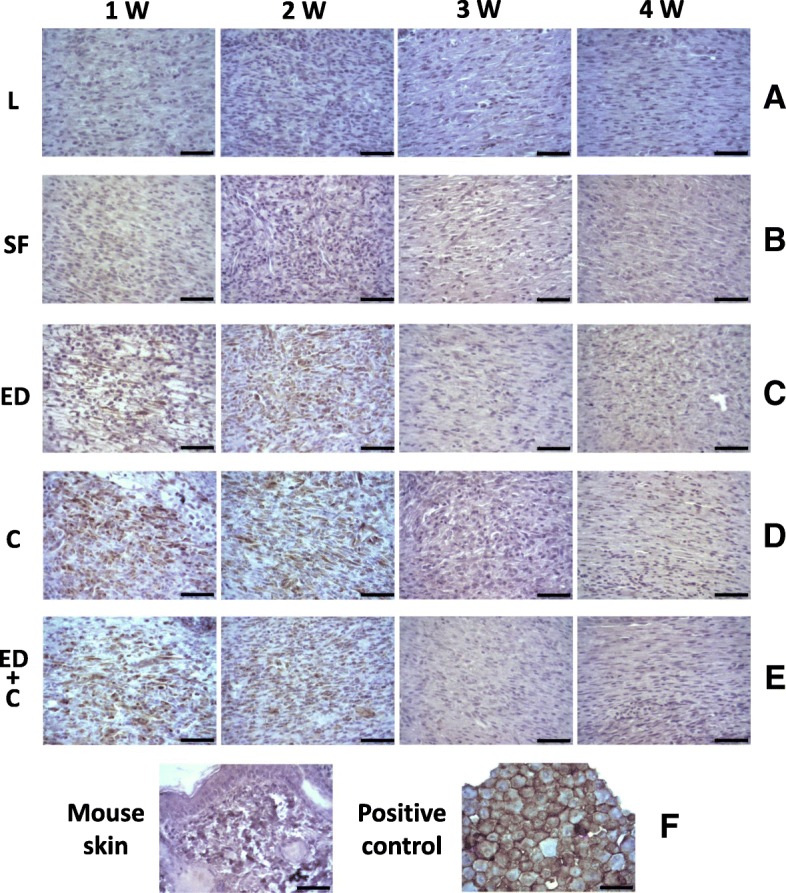
**a**–**e** Expression of the human mesenchymal stem cell marker CD90 on mouse dermal wounds. Experimental groups: *L* (wounds covered with Linitul), *SF* (wounds covered with SF scaffolds), *ED* (wounds with Wj-MSCs injected at the edge), *C* (wounds covered with SF patches cellularized with Wj-MSCs), and *ED+C* (wounds with SF patches cellularized with Wj-MSCs over the wound bed and Wj-MSCs injected at the edge). Immunostaining with the anti-human CD90 antibody of a mouse skin section and a pellet made of Wj-MSCs served as negative and positive controls, respectively (**f**). Scale bar 50 μm

Regarding stem cell proliferation, immunohistochemical staining of human CD90 was absent on normal mouse skin (Fig. 7f, left), Linitul, or SF scaffold-treated wounds (Fig. 7a, b). On the other hand, numerous human CD90-positive cells were observed within the granulation tissue of wounds of all the Wj-MSC-treated groups for the first 2 weeks after injury, being the immunostaining negative from the second week onwards (Fig. 7c–e). Additionally, human CD90-positive cells were also observed in the epithelial front and even formed small nests in the epidermal layer (Additional file 5: Figure S4A-D).

To determine if Wj-MSC had the capacity to transdifferentiate into keratinocyte-like cells, we performed immunohistochemical analysis of different wound sections of Wj-MSCs-SF+Edge-treated animals using a specific anti-human pan cytokeratin antibody (Additional file 6: Figure S5A-F). As reported previously for MSCs isolated from other sources (i.e., adipose tissue or bone marrow) [30, 31], multiple human pan cytokeratin-positive cells were detected at the migrating epithelial front of wounds after 2 weeks of treatment, a result which suggests that Wj-MSCs may also transdifferentiate in vivo*.*

### Histopathological examination of distant organs and analysis of tumor formation after transplantation of Wj-MSCs

In vivo toxicity and biodistribution of Wj-MSCs was evaluated in SKH1 mice to rule out ectopic tissue formation and tumorigenesis. The main histopathological secondary events observed during the healing process were a mild interstitial innate inflammatory infiltrate in the lungs from Wj-MSC-treated groups at 2 and 4 weeks after treatment (Additional file 7: Figure S6A, B), some sporadic hepatic microabscesses in the same groups at 2 weeks post-treatment (Additional file 7: Figure S6C, D), and a certain degree of hyperplasia of splenic pulp during the third and fourth weeks in all experimental groups and Wj-MSC-treated groups, respectively (Additional file 7: Figure S6E, F). Remarkably, no signs of tumors or organomegalies were observed in any examined organ (lungs, heart, liver, spleen, kidneys, testis, brain, and bone marrow) during all the study (Additional file 8: Figure S7A-H). Lastly, the analysis of biodistribution of transplanted Wj-MSCs by human CD90 expression showed no presence of Wj-MSCs in any examined organs apart from the skin (not shown).

## Discussion

Several reports on different animal models and clinical trials indicate that MSCs may play a beneficial role in improving wound healing [32, 33]. In this work, we have isolated from the Wharton’s jelly of the human umbilical cord cells that exhibited the morphology and surface markers typical of MSCs. These cells were significantly more proliferative compared to BM-MSCs, probably due to their primitive nature [34], and they also potently suppressed the proliferation of activated T cells. This finding has clinical implications since allogeneic MSC transplantation might promote immune tolerance [35, 36].

The optimal method of cell delivery is an unsolved issue in cell-based regenerative medicine applications. In this sense, the use of SF three-dimensional scaffolds has shown good biocompatibility, slow and controllable degradability, and minimal inflammatory response and has been used in a variety of configurations in tissue engineering approaches, including cartilage regeneration, corneal repair, and wound dressing [37–39].

We tested if electrospun SF combined with Wj-MSCs would work synergistically improving its therapeutic effect on wound healing as compared to each treatment alone. Scanning electron microscopy micrographs of Wj-MSCs growing on SF scaffold show that Wj-MSCs adhered to the SF fibers and stretched across the nanofibrous substrates during their proliferation. Also, they crosslinked the nanofibers and integrated with the surrounding fibers to form a three-dimensional cellular network similar to that of the natural extracellular matrix found in the normal skin, even also observing that Wj-MSCs in contact with the SF scaffold could be able to express some level of collagen, the main structural protein of connective tissue forming the dermis. Finally, this artificial “dermal layer” adheres to and integrates well within the wound bed. Regarding wound repair, although the use of Wj-MSC injected at the wound edge accelerated the formation of the epithelial sheet, the combined therapy of Wj-MSC on the SF scaffold and also injected at the wound edge showed a synergistic effect and improved the quality of the scar, giving rise to histological structures similar to the normal skin.

Previous studies have shown that human umbilical cord MSCs could enhance the healing of mouse skin wounds when administered locally by increasing re-epithelialization and cellularity and by differentiating directly into epithelial cells expressing keratin [40, 41]. Our findings of the presence of CD90-positive cell nests on the epithelial sheet in the wound of mice treated with Wj-MSCs-SF+Edge at day + 14 are in agreement with these previous observations. Moreover, the presence of specific human CD90 and cytokeratin-positive spindle-shaped cells within granulation tissue suggests that Wj-MSCs may not only transdifferentiate into specialized skin cells but also participate in the healing process through other indirect mechanisms.

The transition from the inflammatory to the cell proliferation phase during wound healing is a key step during this process. Prolonged inflammation is detrimental and may induce keratinocyte proliferation deregulation and excessive scarring [42, 43]. Thus, we aimed to study whether the immunoregulatory properties that characterized Wj-MSCs in vitro had a biological effect on wound healing. The epithelial sheet regeneration in our model is usually completed in 2 weeks, as demonstrated by the H&E stain of wound tissues in mice treated with Linitul. When the SF scaffold was present in the wound bed, maturation of granulation tissue and re-epithelialization were delayed about a week. Thus, the presence of the SF scaffold seemed to enhance a prolonged innate immune response of the body against the biomaterial that would explain the temporary presence of a greater number of PMN neutrophils in the wound tissue. However, once the Wj-MSCs were transplanted to the wound site either associated with the SF scaffold or injected at the wound edge, the immune allo-response decreased, as evidenced by the low number of PMN neutrophils and macrophages at the wound site of mice treated with Wj-MSCs. This local modulation of the cellular inflammatory response might be beneficial for tissue repair since it is associated with reduced scar formation and the replacement of damaged tissue with the functional skin. This study corroborated previous findings that human MSCs elicit little immunogenicity from their xenogeneic host indicating that MSCs are immunoprivileged during cutaneous wound healing [44, 45].

Our results indicate that Wj-MSCs exert an immunomodulatory role over the innate immune response during the initial inflammatory phase of wound healing. This influence does not involve adaptative T cell-mediated immunity, as demonstrated by the noteworthy existence of T CD3-positive cells during the first 2 weeks of healing. However, further investigations are needed to elucidate the cell interactions between Wj-MSCs and immune cells involved in the wound healing process, to optimize the timing and dosage of MSC delivery to the wound for maximal efficacy.

Our data also suggest that promotion of angiogenesis is one of the mechanisms by which Wj-MSCs enhanced wound healing in SKH1 mice as reported by other groups [46, 47]. This fact was corroborated by the higher vascularized area observed in wounds that underwent MSC therapy as compared with experimental treatments that contain the SF scaffold alone. Although the SF scaffold per se displayed an early increase in vascularization of the wound, this effect was transient and only was maintained throughout the healing process mainly when MSCs were in association with SF. These results emphasize the importance of the MSC-based scaffolds instead of the MSC therapy alone in improving the proliferation and migration of blood vessels within the biomaterial and the granulation tissue [32, 48].

The key cellular mediator of fibrosis is the myofibroblast that is a primary collagen-producing cell and promoter of wound contraction. In our study, α-SMA and desmin, both markers of distinct myofibroblast subpopulations, were strongly detected during the first 2 weeks of healing of the group treated with the SF scaffold and the group treated with Wj-MSCs injected around the wound margins. By contrast, when applied together, Wj-MSCs and the SF scaffold, mainly the group Wj-MSCs-SF+Edge, we observed less α-SMA and desmin-positive cell expression and a scarce arrangement of the collagen (similar to normal skin collagen distribution). This data suggest that Wj-MSCs in association with SF might act in synergy and promote the production of an ECM that more closely resembles uninjured dermal tissue. Similarly, Huang et al. found that wounds treated with MSCs had reduced the thickness of collagen fibrils that were well organized in a basket-weave pattern after 14 days. Also, the expression of type I collagen and α-SMA was downregulated in MSC treatment groups and significantly decreased in wounds with MSCs delivered by microspheres [49].

Importantly, histopathological examination of distant organs of mice receiving human Wj-MSCs did not experience abnormal proliferation or formed teratomas and/or tumors. Analysis of biodistribution of transplanted Wj-MSCs did not show the presence of the cells in any examined organs. Thus, xenotransplant of human Wj-MSCs combined with SF scaffolds in SKH1 mice proved to be a safe and effective therapeutic alternative for the treatment of skin wounds.

## Conclusions

Taken together, our data provide evidence that the combined treatment of Wj-MSCs injected at the wound edge along with cellularized SF scaffolds covering the wound site exhibited better wound healing capabilities as compared with both single treatments and the cellularized SF scaffold. Cell therapy with Wj-MSC-based SF construct contributed to the generation of a high-quality, well-vascularized granulation tissue; enhanced re-epithelialization of the wound; and attenuated the formation of fibrotic scar tissue by decreasing myofibroblast proliferation. Although more studies are necessary to understand the mechanistics of the Wj-MSC beneficial function, cell therapy with Wj-MSCs might be a safe alternative to improve healing of skin wounds refractory to standard therapy.

## Additional files

Additional file 1: Supplemental Methods text. Description of methods for the characterization of physical and mechanical properties of cellularized electrospun silk fibroin scaffolds. (DOCX 13 kb)

Additional file 2: **Figure S1.** SEM micrographs (A), histogram of fiber diameters (B), and examples of stress-elongation curves (C) of electrospun silk fibroin mats produced with 19% regenerated silk fibroin solutions. (PDF 77 kb)

Additional file 3: **Figure S2.** Viability of Wj-MSCs cultured on electrospun SF scaffolds for different times. (A) Percentages of Annexin-V^−^/7-AAD^−^ cells were analyzed by flow cytometry. (B) Percentage of viable cells significantly decreased compared to values obtained at time 0 (*p < 0.05, **p < 0.01), according to one-way ANOVA. Also, percentages of viable cells significantly decreased after culture on SF patches compared to the control group at each time point (^#^*p* < 0.05, ^###^*p* < 0.001). Results are shown as mean ± SD of three independent experiments performed in triplicates. (PDF 447 kb)

Additional file 4: **Figure S3.** Masson’s trichrome stain of electrospun silk fibroin scaffold seeded with Wharton’s jelly mesenchymal stem cells at a density of 5 × 10^4^ cells/cm^2^ for 4 days. A: × 100 magnification. B: × 200 magnification. Scale bar 100 μm. (PDF 475 kb)

Additional file 5: **Figure S4.** Expression at 2 weeks of the human mesenchymal stem cell marker CD90 at the migrating epithelial front in wounds from mice treated with silk fibroin patches cellularized with Wj-MSCs and Wj-MSCs injected at the edge of the wound. Scale bar 100 μm (A) and 20 μm (B–D). (PDF 621 kb)

Additional file 6: **Figure S5.** Analysis of transdifferentiation of Wj-MSCs into keratinocyte-like cells. Expression of human pan cytokeratin was analyzed by immunohistochemistry at the migrating epithelial front of wounds from mice after 2 weeks of treatment with silk fibroin patches cellularized with Wj-MSCs plus Wj-MSCs injected at the edge of the wound. Human (E) or mouse (F) skin sections were used as positive and negative controls, respectively. Scale bar 100 μm (A, C, E, F) and 50 μm (B, D). (PDF 389 kb)

Additional file 7: **Figure S6.** Histopathological findings in the lung (A, B), liver (C, D), and spleen (E, F) sections stained by H&E. (A, B) Interstitial inflammatory infiltrate (#), and alveolar macrophages (<) in the lung tissue. (C, D) Image of a liver microabscess (§), with polymorphonuclear cells in it (*). (E, F) Hyperplasia of the splenic white pulp (+: splenic arteriole). Abbreviations: wp, white pulp; rp, red pulp. Left panels: × 20 magnification. Right panels: × 40 magnification. (PDF 423 kb)

Additional file 8: **Figure S7.** H&E staining for standard histological examination of the (A) lung, (B) heart, (C) liver, (D) spleen, (E) kidney, (F) testis, (G) brain and (H) bone marrow. Scale bar 50 μm. (PDF 388 kb)

## Abbreviations

7-AAD: 7-Amino-actinomycin
BM: Bone marrow
BM-MSCs: Bone marrow mesenchymal stem cells
DMEM: Dulbecco’s modified Eagle’s medium
ECM: Extracellular matrix
H&E: Hematoxylin and eosin
HPF: High-power fields
IDO: Indoleamine 2,3-dioxygenase
MNCs: Mononuclear cells
MSCs: Mesenchymal stem cells
PD: Population doubling level
PDT: Population doubling time
PGE_2_: Prostaglandin E_2_
PMN: Polymorphonuclear neutrophil
SEM: Scanning electron microscopy
SF: Silk fibroin
TGF-β: Transforming growth factor beta
TSG-6: Tumor necrosis factor-stimulated gene 6 protein
Wj-MSCs: Wharton’s jelly mesenchymal stem cells
α-SMA: Alpha-smooth muscle actin
