# Supplementary material for: Silk fibroin scaffolds seeded with Wharton’s jelly mesenchymal stem cells enhance re-epithelialization and reduce formation of scar tissue after cutaneous wound healing
Source: Stem Cell Res Ther. 2019 Apr 27;10:126. doi: 10.1186/s13287-019-1229-6 (PMC6487033; doi:10.1186/s13287-019-1229-6)
Supplement: Supplementary file 1 — Supplemental Methods text. Description of methods for the characterization of physical and mechanical properties of cellularized electrospun silk fibroin scaffolds. (DOCX 13 kb) [file 13287_2019_1229_MOESM1_ESM.docx]

**Supplemental Methods**

**Characterization of physical and mechanical properties of cellularized electrospun silk fibroin scaffolds**

Electrospun silk fibroin scaffolds was elaborated as previously reported [1]. Conformational and structural analysis of electrospun SF scaffolds before and after seeding with hWj-MSCs was performed by scanning electron microscopy (SEM). The SF patches were fixed with 3% glutaraldehyde in 0.1M cacodylate buffer for 1.5h at 4°C, and then they were rinsed and post-fixed in OsO_4_ for 1h. Subsequently, they were dehydrated by washing with solutions of increasing concentrations of ethanol. Finally, the samples were placed on metal stubs, dried by the critical point method and gold coated. The meshes were visualized using a Jeol T-6100 scanning electronic microscope (SEAL Labs) at 15 kV, after being sputter-coated with gold. The pictures obtained were used to determine the maximum transverse size of the electrospun fibers using the ImageJ software.

To evaluate mechanical properties of electrospun SF scaffolds tensile tests were performed using a universal test frame machine (Qtest, MTS Systems). The mechanical properties of specimens (10 mm x 30 mm) were recorded with a crosshead speed of 0.1 mm/s and a load cell of 5 N, under ambient conditions. The thickness of each piece of SF scaffold was determined with a Mitutoyo Digimatic Micrometer (Mitutoyo America Corporation) 0-25 mm, with a resolution of 0.001 mm and an accuracy of ±2 mm. Young’s modulus (MPa), ultimate strength (MPa), and elongation at rupture (%) were determined using the stress–strain curves. Young’s modulus was calculated in the linear elastic portion of stress-strain curves generated. Each test was performed at least four times per scaffold.

**SUPPLEMENTAL REFERENCES**

1. Aznar-Cervantes S, Roca MI, Martinez JG, Meseguer-Olmo L, Cenis JL, Moraleda JM, Otero TF. Fabrication of conductive electrospun silk fibroin scaffolds by coating with polypyrrole for biomedical applications. Bioelectrochemistry. 2012; 85: 36-43.
