# Supplementary material for: Silk fibroin scaffolds seeded with Wharton’s jelly mesenchymal stem cells enhance re-epithelialization and reduce formation of scar tissue after cutaneous wound healing
Source: Stem Cell Res Ther. 2019 Apr 27;10:126. doi: 10.1186/s13287-019-1229-6 (PMC6487033; doi:10.1186/s13287-019-1229-6)
Supplement: Supplementary file 2 — Figure S1. SEM micrographs (A), histogram of fiber diameters (B), and examples of stress-elongation curves (C) of electrospun silk fibroin mats produced with 19% regenerated silk fibroin solutions. (PDF 77 kb) [file 13287_2019_1229_MOESM2_ESM.pdf]

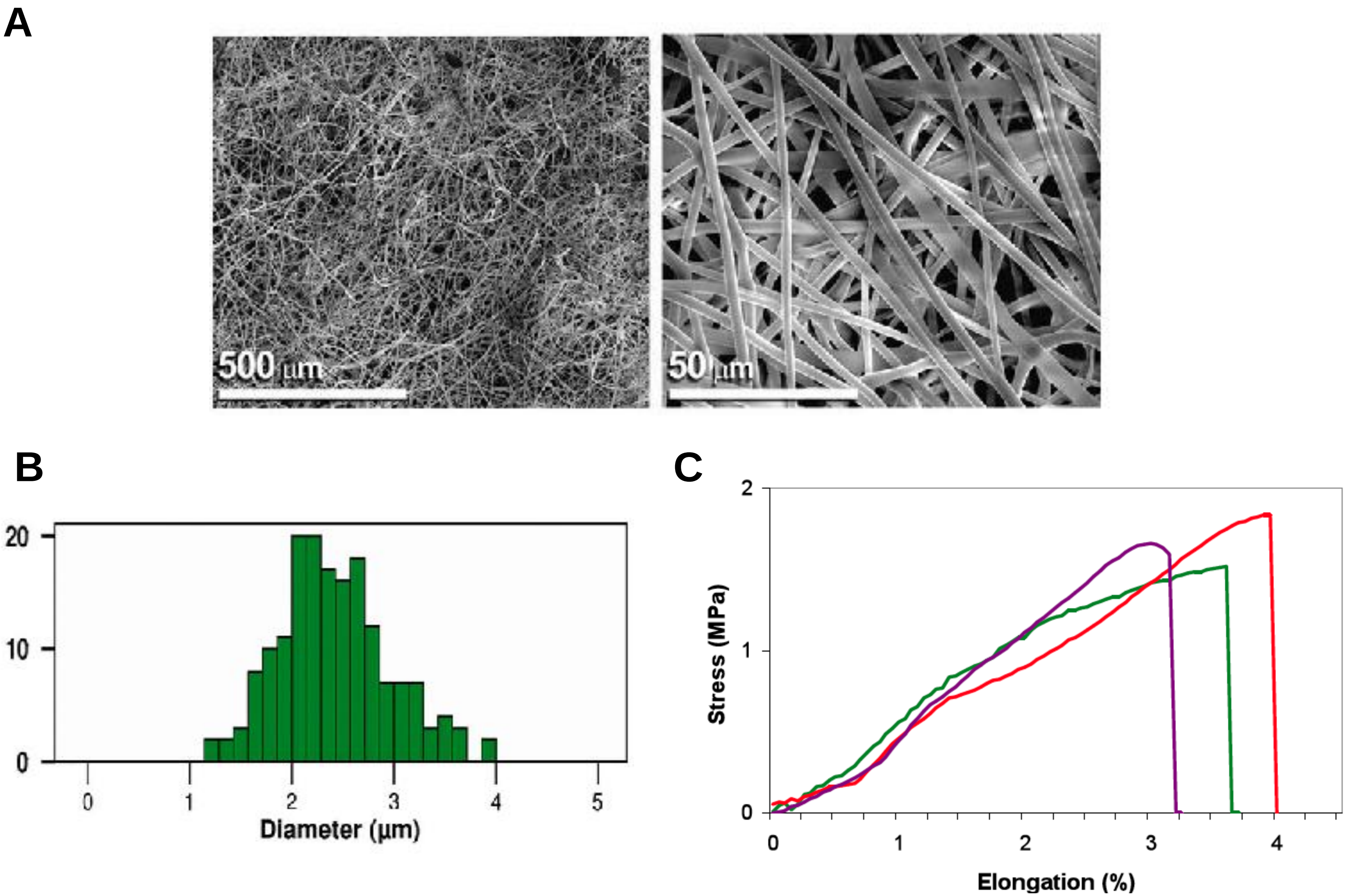

Fig. S1. SEM micrographs **(A)**, histogram of fiber diameters **(B)** and examples of stress-elongation curves **(C)** of electrospun silk fibroin mats produced with 19% regenerated silk fibroin solutions.
