# Supplementary material for: Silk fibroin scaffolds seeded with Wharton’s jelly mesenchymal stem cells enhance re-epithelialization and reduce formation of scar tissue after cutaneous wound healing
Source: Stem Cell Res Ther. 2019 Apr 27;10:126. doi: 10.1186/s13287-019-1229-6 (PMC6487033; doi:10.1186/s13287-019-1229-6)
Supplement: Supplementary file 3 — Figure S2. Viability of Wj-MSCs cultured on electrospun SF scaffolds for different times. (A) Percentages of Annexin-V−/7-AAD− cells were analyzed by flow cytometry. (B) Percentage of viable cells significantly decreased compared to values obtained at time 0 (*p < 0.05, **p < 0.01), according to one-way ANOVA. Also, percentages of viable cells significantly decreased after culture on SF patches compared to the control group at each time point (#p < 0.05, ###p < 0.001). Results are shown as mean ± SD of three independent experiments performed in triplicates. (PDF 447 kb) [file 13287_2019_1229_MOESM3_ESM.pdf]

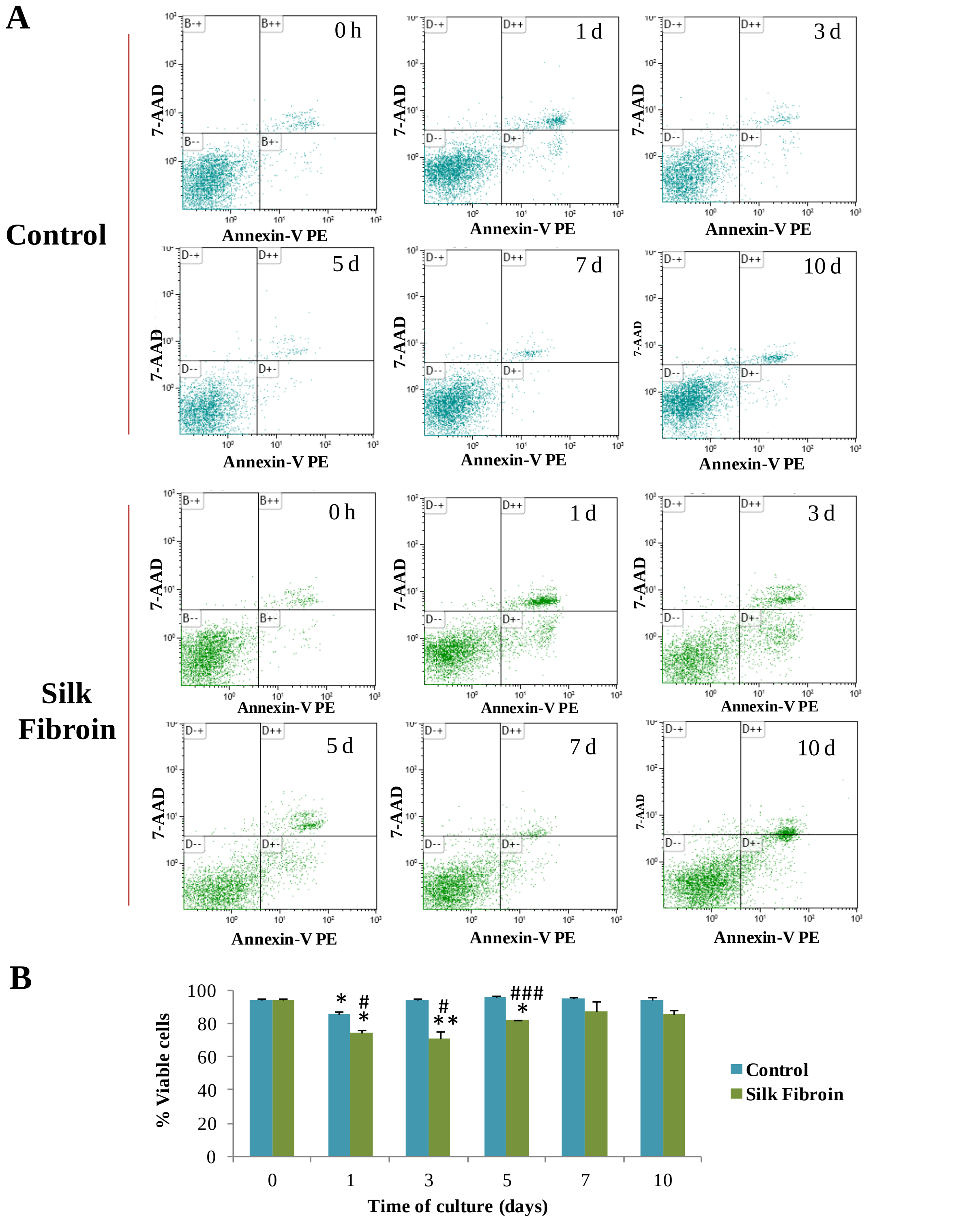

Fig. S2. Viability of Wj-MSCs cultured on electrospun SF scaffolds for different times. **(A)** Percentages of Annexin-V⁻/7-AAD⁻ cells were analyzed by flow cytometry. **(B)** Percentage of viable cells significantly decreased compared to values obtained at time 0 (*p<0.05, **p<0.01), according to one-way ANOVA. Also, percentages of viable cells significantly decreased after culture on SF patches compared to the control group at each time point ($^{\#}p$<0.05, $^{\#\#\#}p$<0.001). Results are shown as mean ± SD of three independent experiments performed in triplicates.
