# Supplementary material for: Silk fibroin scaffolds seeded with Wharton’s jelly mesenchymal stem cells enhance re-epithelialization and reduce formation of scar tissue after cutaneous wound healing
Source: Stem Cell Res Ther. 2019 Apr 27;10:126. doi: 10.1186/s13287-019-1229-6 (PMC6487033; doi:10.1186/s13287-019-1229-6)
Supplement: Supplementary file 4 — Figure S3. Masson’s trichrome stain of electrospun silk fibroin scaffold seeded with Wharton’s jelly mesenchymal stem cells at a density of 5 × 10^4^ cells/cm2 for 4 days. A: × 100 magnification. B: × 200 magnification. Scale bar 100 μm. (PDF 475 kb) [file 13287_2019_1229_MOESM4_ESM.pdf]

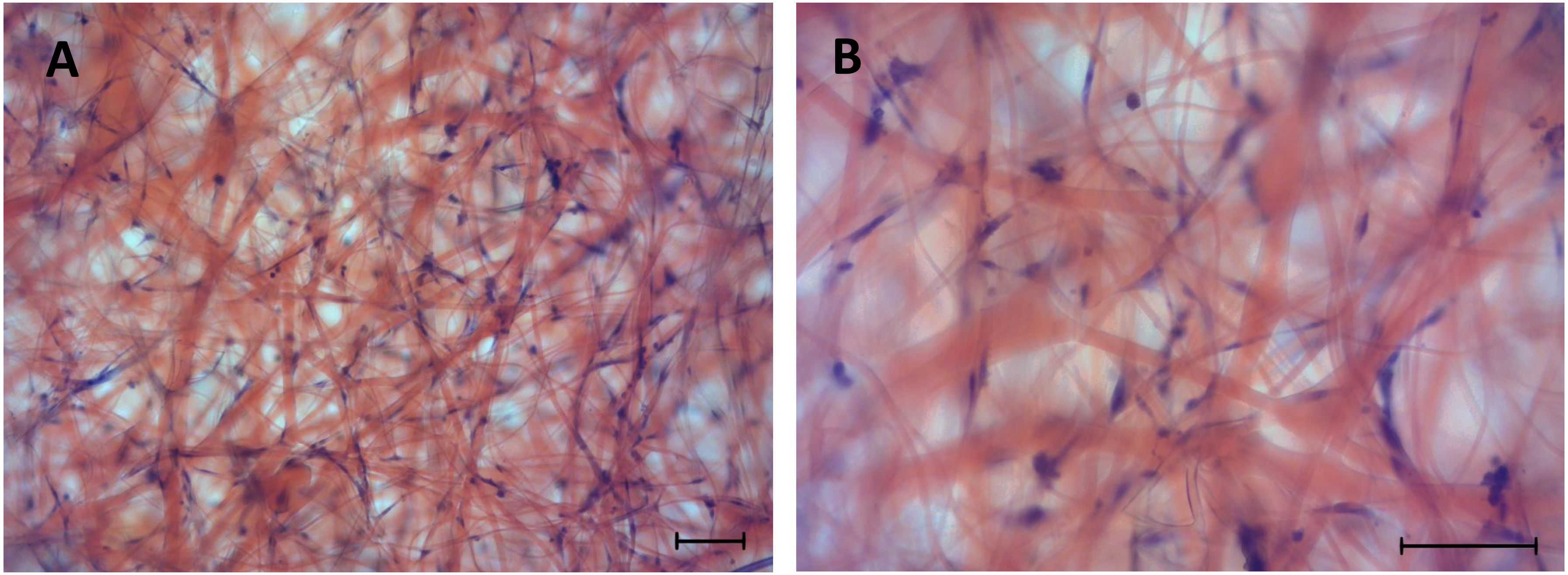

Fig. S3. Masson's trichrome stain of electrospun silk fibroin scaffold seeded with Wharton's jelly mesenchymal stem cells at a density of $5x10^4$ cells/cm^2 for four days. A: 100X magnification. B: 200X magnification. Scale bar: 100 µm.
