# Supplementary material for: Silk fibroin scaffolds seeded with Wharton’s jelly mesenchymal stem cells enhance re-epithelialization and reduce formation of scar tissue after cutaneous wound healing
Source: Stem Cell Res Ther. 2019 Apr 27;10:126. doi: 10.1186/s13287-019-1229-6 (PMC6487033; doi:10.1186/s13287-019-1229-6)
Supplement: Supplementary file 5 — Figure S4. Expression at 2 weeks of the human mesenchymal stem cell marker CD90 at the migrating epithelial front in wounds from mice treated with silk fibroin patches cellularized with Wj-MSCs and Wj-MSCs injected at the edge of the wound. Scale bar 100 μm (A) and 20 μm (B–D). (PDF 621 kb) [file 13287_2019_1229_MOESM5_ESM.pdf]

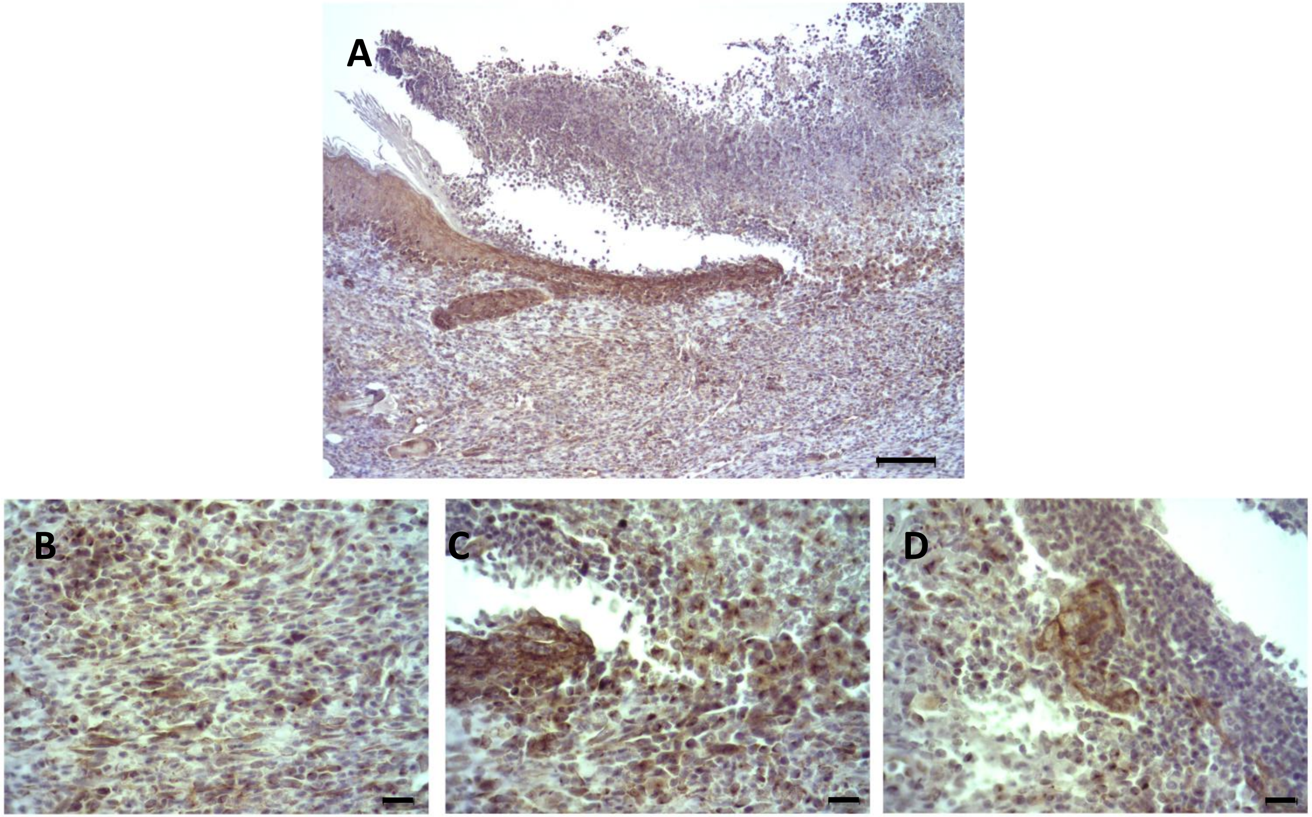

Fig. S4. Expression at 2 weeks of the human mesenchymal stem cell marker CD90 at the migrating epithelial front in wounds from mice treated with silk fibroin patches cellularized with Wj-MSCs and Wj-MSCs injected at the edge of the wound. Scale bar: 100 μm (**A**) and 20 μm (**B-D**).
