# Supplementary material for: Silk fibroin scaffolds seeded with Wharton’s jelly mesenchymal stem cells enhance re-epithelialization and reduce formation of scar tissue after cutaneous wound healing
Source: Stem Cell Res Ther. 2019 Apr 27;10:126. doi: 10.1186/s13287-019-1229-6 (PMC6487033; doi:10.1186/s13287-019-1229-6)
Supplement: Supplementary file 6 — Figure S5. Analysis of transdifferentiation of Wj-MSCs into keratinocyte-like cells. Expression of human pan cytokeratin was analyzed by immunohistochemistry at the migrating epithelial front of wounds from mice after 2 weeks of treatment with silk fibroin patches cellularized with Wj-MSCs plus Wj-MSCs injected at the edge of the wound. Human (E) or mouse (F) skin sections were used as positive and negative controls, respectively. Scale bar 100 μm (A, C, E, F) and 50 μm (B, D). (PDF 389 kb) [file 13287_2019_1229_MOESM6_ESM.pdf]

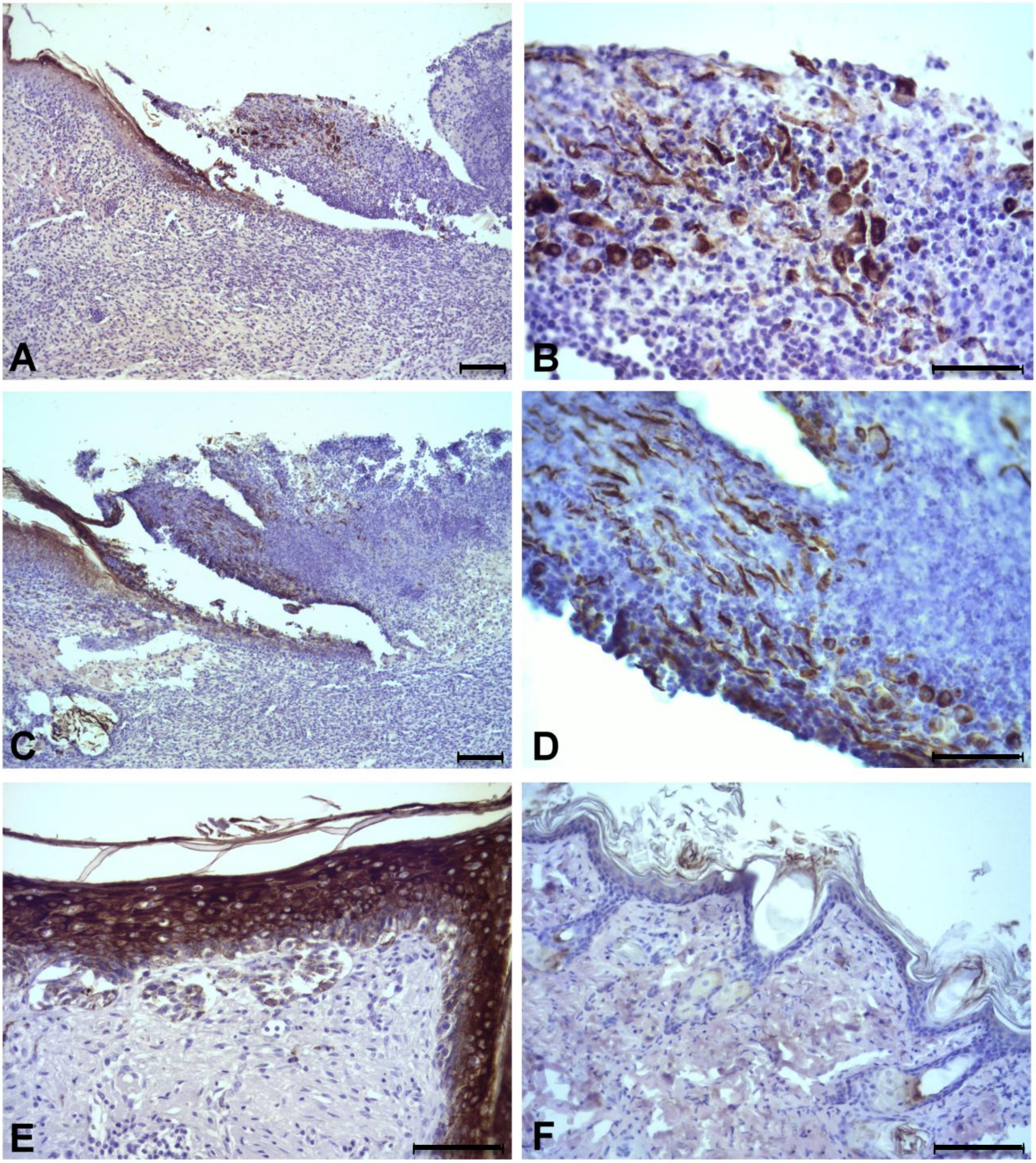

Fig. S5. Analysis of transdifferentiation of Wj-MSCs into keratinocyte-like cells. Expression of human pan cytokeratin was analyzed by immunohistochemistry at the migrating epithelial front of wounds from mice after 2 weeks of treatment with silk fibroin patches cellularized with Wj-MSCs plus Wj-MSCs injected at the edge of the wound. Human **(E)** or mouse **(F)** skin sections were used as positive and negative controls, respectively. Scale bar: 100 µm (**A, C, E, F**) and 50 µm (**B, D**).
