# Supplementary material for: Silk fibroin scaffolds seeded with Wharton’s jelly mesenchymal stem cells enhance re-epithelialization and reduce formation of scar tissue after cutaneous wound healing
Source: Stem Cell Res Ther. 2019 Apr 27;10:126. doi: 10.1186/s13287-019-1229-6 (PMC6487033; doi:10.1186/s13287-019-1229-6)
Supplement: Supplementary file 7 — Figure S6. Histopathological findings in the lung (A, B), liver (C, D), and spleen (E, F) sections stained by H&E. (A, B) Interstitial inflammatory infiltrate (#), and alveolar macrophages (<) in the lung tissue. (C, D) Image of a liver microabscess (§), with polymorphonuclear cells in it (*). (E, F) Hyperplasia of the splenic white pulp (+: splenic arteriole). Abbreviations: wp, white pulp; rp, red pulp. Left panels: × 20 magnification. Right panels: × 40 magnification. (PDF 423 kb) [file 13287_2019_1229_MOESM7_ESM.pdf]

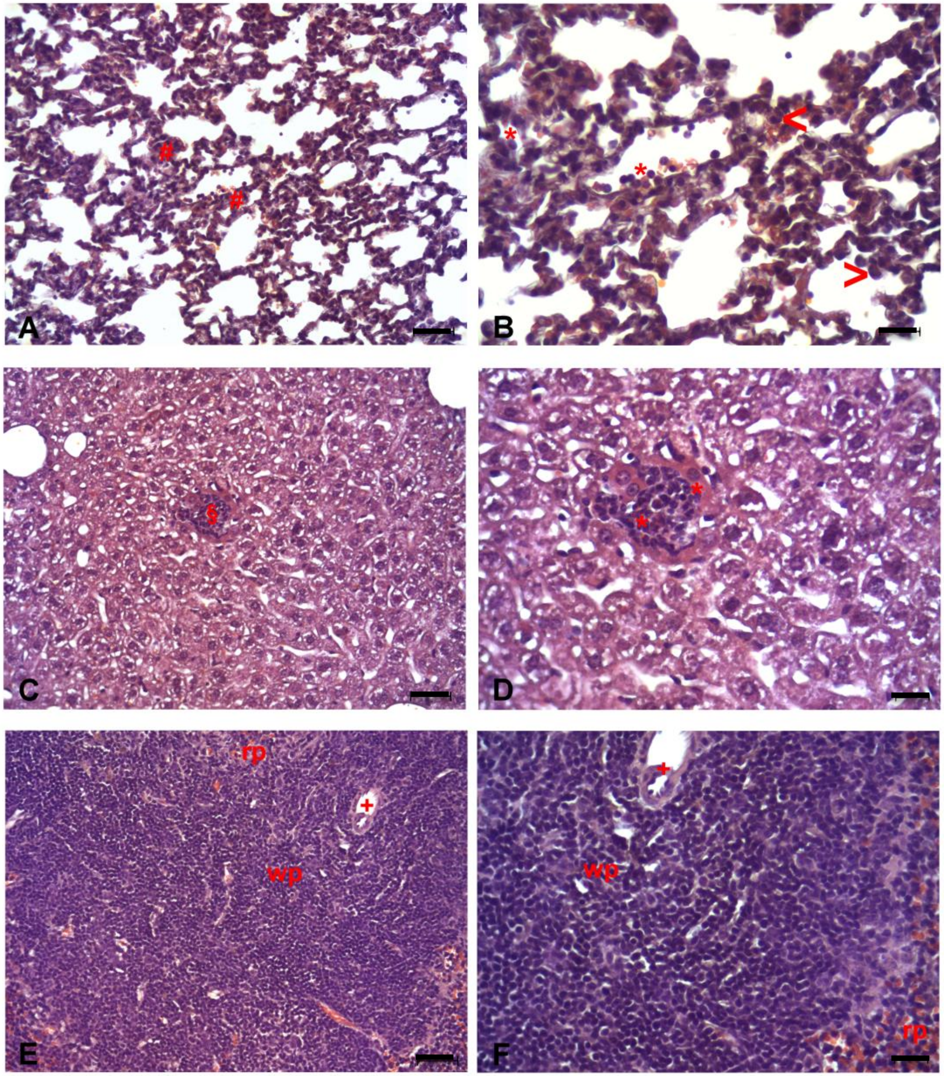

Fig. S6. Histopathological findings in lung (**A, B**), liver (**C, D**), and spleen (**E, F**) sections stained by H&E. (**A, B**) Interstitial inflammatory infiltrate (**#**), and alveolar macrophages (**<**) in lung tissue. **(C, D)** Image of a liver microabscess (**§**), with polymorphonuclear cells in it (*****). **(E, F)** Hyperplasia of the splenic white pulp (**+**: splenic arteriole). Abbreviations: **wp**: white pulp; **rp**: red pulp. Left panels: 20X magnification. Right panels: 40X magnification.
