# Supplementary material for: Silk fibroin scaffolds seeded with Wharton’s jelly mesenchymal stem cells enhance re-epithelialization and reduce formation of scar tissue after cutaneous wound healing
Source: Stem Cell Res Ther. 2019 Apr 27;10:126. doi: 10.1186/s13287-019-1229-6 (PMC6487033; doi:10.1186/s13287-019-1229-6)
Supplement: Supplementary file 8 — Figure S7. H&E staining for standard histological examination of the (A) lung, (B) heart, (C) liver, (D) spleen, (E) kidney, (F) testis, (G) brain and (H) bone marrow. Scale bar 50 μm. (PDF 388 kb) [file 13287_2019_1229_MOESM8_ESM.pdf]

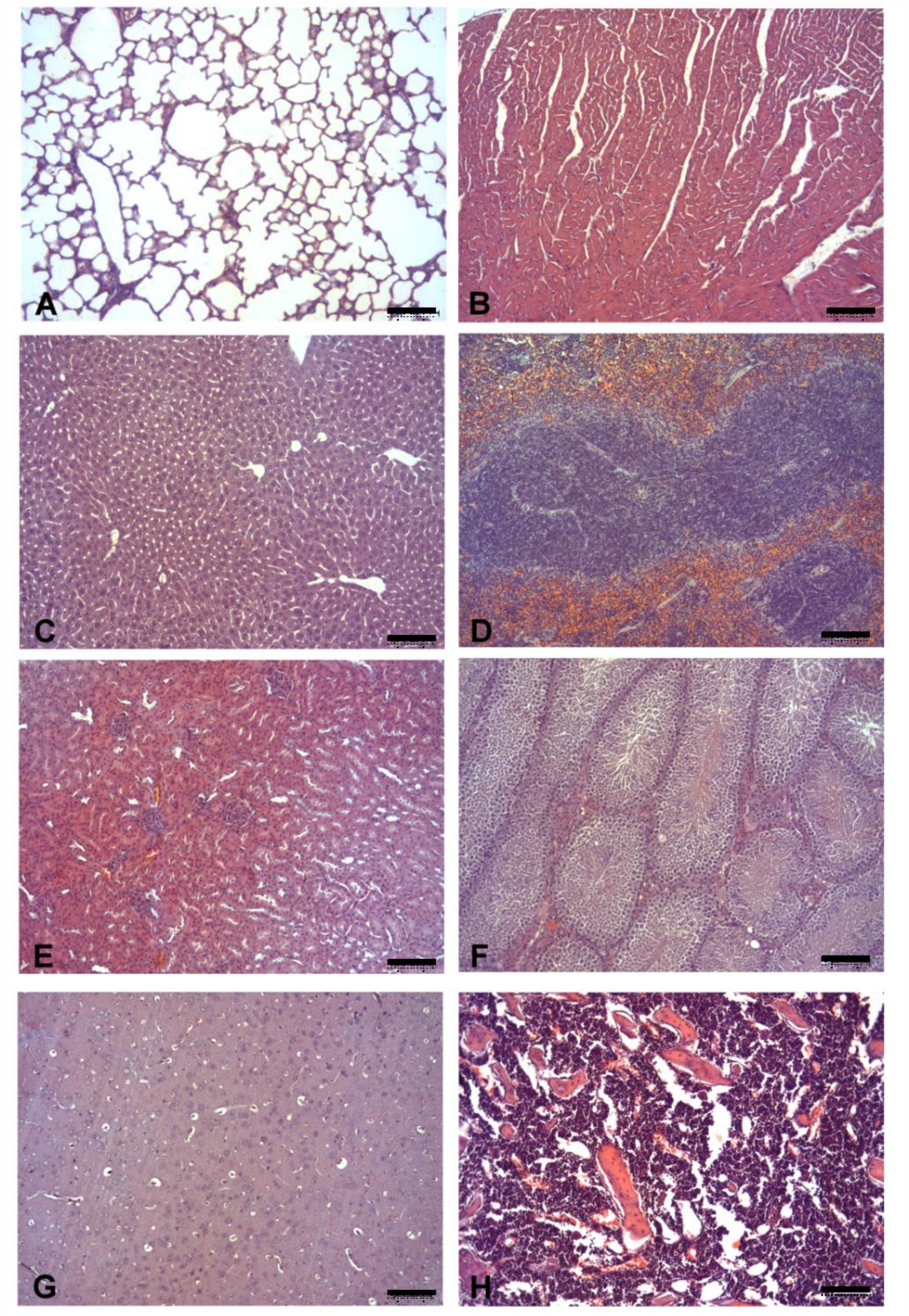

Fig. S7. H&E staining for standard histological examination of **(A)** lungs, **(B)** heart, **(C)** liver, **(D)** spleen, **(E)** kidney, **(F)** testis, **(G)** brain and **(H)** bone marrow. Scale bar: 50 µm.
